# The effect of lumbar spinal manipulation on biomechanical factors and perceived transient pain during prolonged sitting: a laboratory-controlled cross-sectional study

**DOI:** 10.1186/s12998-022-00472-y

**Published:** 2022-12-30

**Authors:** D. E. De Carvalho, J. P. Callaghan

**Affiliations:** 1 Faculty of Medicine, Memorial University of Newfoundland, St. John’s, NL A1B 3V6 Canada; 2 Department of Kinesiology & Health Sciences, Faculty of Health, University of Waterloo, Waterloo, ON N2L 3G1 Canada

**Keywords:** Sitting, Low back, Spine posture, Spine movements, Manipulation

## Abstract

**Background:**

Spinal manipulation has been shown to affect muscle activity, posture, and pain. To date, no studies have examined the effect of manipulation on biomechanical factors during sitting. Therefore, the purpose of this study was to investigate the immediate effect of lumbar spinal manipulation on trunk muscle activation, spine posture and movements, and perceived ratings of transient pain in asymptomatic adults during prolonged office sitting.

**Methods:**

Twenty healthy adults were recruited for a single laboratory session that included a standardized office sitting/data entry protocol (120 min total, 3 blocks of 40 min). Data were collected between July and August 2012. The first block (baseline) was immediately followed by two experimental blocks. Prior to the start of each experimental block, participants were transferred to a therapy plinth and placed side lying (right side down), and a random presentation of either a control or high velocity low amplitude thrust directed at L4/L5 was delivered. Continuous measures of muscle activity, spine posture, and spine movements were recorded throughout the sitting trials. Perceived transient pain was measured by visual analogue scale at 10-min intervals (including immediately before and after the randomized maneuvers).

**Results:**

There were no significant differences in spine or pelvic posture or perceived back pain following either the manipulation or control maneuvers. Significantly reduced muscle activity and increased shifts of the lumbar spine angle were identified in the block following manipulation compared to both baseline and post control blocks.

**Conclusions:**

Spinal manipulation does not appear to have an immediate impact on spine or pelvic posture in healthy adults but does appear to reduce muscle activity and increase spine movement during sitting. Future work should replicate this study with a larger population in a field setting. It may be worthwhile to explore the implication of reduced muscle activation and increased spine movements during prolonged sitting for office workers that receive manipulations or mobilizations during their workday.

## Introduction

Moderate evidence supports manipulation of spinal facet joints for the treatment of acute and chronic low back pain [1–3]. Spinal manipulative therapy is also utilized by patients as maintenance care between symptomatic episodes [4] and occasionally by asymptomatic individuals for wellness/preventative care [5]. The responses most connected to the therapeutic benefit of spinal manipulation include reduced pain [6–12], increased range of motion [10, 13], altered muscle activation [9, 10, 14–20], increased postural awareness [21–26], and improved performance of functional movements [13]. Despite these demonstrated treatment effects, the exact mechanisms behind them have remained hypothetical. Mechanisms thought to be involved include reflex pathways from muscle spindle, Golgi-tendon and mechanoreceptors in the facet joint capsules, ligaments, deep spine muscles and overlying skin of the back that are activated respectively during the thrust phase of the manipulation procedure [20, 27].

There are two key knowledge gaps pertaining to the effect of manipulation on muscle activity and lumbar spine posture; both of which are relevant to sitting. Considering the potential association to low back pain and the effect posture and muscle activity play during sitting, and given the not uncommon scenario of office workers being treated at some point during their workday (e.g., onsite clinics, appointments during lunch breaks etc.), it would be helpful to explore the potential effects of spinal manipulative therapy (SMT) on these parameters during sitting. Specifically, considering the evidence of reduced lumbar paraspinal muscle activity in forward trunk flexion [14, 17] after manipulation and the hypothesized factor of sustained low-grade muscle activity in the generation of discomfort during sitting [28], perhaps this intervention has a role in improving the effects of seated exposures. Similarly, altered kinesthetic awareness has been shown to decrease in the lumbar spine in response to prolonged flexion [29]. Preliminary investigations of manipulation in chronic neck pain patients have found significant improvements in head repositioning ability [25], neck posture [23], and elbow-repositioning ability [21]. While there is evidence that lumbar repositioning is adversely affected in low back pain (LBP) patients [30], the effect of SMT on balance and postural awareness in this population has been variable [31, 32], and to date, no studies have examined the immediate effect of manipulation on posture of the lumbar spine in sitting. However, if the response is similar to the cervical spine, perhaps manipulation could effect lumbar posture in sitting by creating more postural awareness. To limit the variability that likely results from the confounding effects of clinical presentation, answering this research question first with a healthy sample is warranted.

### Purpose

The purpose of this study was to investigate the effect of a high velocity low amplitude (HVLA) lumbar spinal manipulation on trunk muscle activation, spine posture and movements during prolonged office sitting. A secondary outcome included perceived ratings of transient pain. It was hypothesized that manipulation would not lead to differences in spine posture, muscle activity or perceived transient pain during the sitting exposure, and that there would be no differences between males and females.

## Methods

### Study design

This is a cross-sectional laboratory-controlled study with a within-group design that was collected between July–August 2012.

### Participants

A convenience sample of participants willing to undergo instrumentation with indwelling electrodes, with no recent (6 month) history of acute low back pain, an episode severe enough to seek treatment or miss school/work, were recruited from a university population. This population was chosen since they would be accustomed to sitting for extended periods of the day and should generally be free from degenerative changes of the spine commonly found in older individuals. We aimed to recruit as many participants as possible within a 3-month window. Informed written consent was completed prior to testing and the study received ethics approval from the Office of Research Ethics at our institution (ORE#17708).

### Instrumentation

#### Muscle activity

Indwelling electromyographic (EMG) data were collected from multifidus bilaterally at L4/L5. Bipolar 44 μm gauge, 10 cm long fine wire nickel alloy electrodes with 2 mm exposed tips bent into hooks (VIASYS Healthcare, Excellence for Life Neurocare Group, Madison, WI, USA), were inserted into the deep multifidus muscle with a 27-gauge hypodermic needle using real-time diagnostic ultrasound imaging for guidance (M-Turbo, Sonosite Inc., Bothell, WA, USA). Specifically, the needle was inserted 10 mm lateral to the midpoint of the spinous process of L4 in a slight craniomedial orientation to a depth approximately 5 mm less than the vertebral lamina [33]. Before the needle was withdrawn, the real-time EMG signal was checked by having the participant raise their ipsilateral leg against mild resistance applied by the researcher [34]. Before continuing, the participant was instructed to contract their muscles a few times while lying prone so any temporary muscle spasms (if present) could settle. Raw EMG signals were band pass filtered from 10 to 2000 Hz, amplified (AMT-8, Bortec, Calgary, Canada: CMRR = 115 db at 60 Hz and input impedance = 10 GΩ) and collected at a sampling rate of 4096 Hz with a 16-bit A/D converter (± 2.5 V range). Maximum voluntary contraction trials were collected with the participant extending against resistance with their torso suspended off the edge of an examination bench. A 5 s resting trial was taken with the participant lying prone. Removal of the electrodes at the end of collection was done under ultrasound guidance to confirm that there was no displacement of the wires during the maneuvers [35].

Surface EMG was also collected. The skin at each electrode site was first prepared by lightly shaving the area and wiping with 70% isopropyl alcohol. A ground electrode was placed on the clavicle. Eight channels of surface EMG were collected using two disposable electrodes (Ag–AgCl, Blue Sensor, Medicotest Inc., Ølstykke, Denmark) with a 2 cm inter-electrode distance and parallel to muscle fiber orientation bilaterally over the thoracic erector spinae (5 cm lateral to the spinous process of T9), lumbar erector spinae (5 cm lateral to spinous process of L1), lumbar multifidus (superiomedial angle, 1 cm lateral from the spinous process of L4 with the indwelling leads centred between the pair) and gluteus medius (2.5 cm distal to the midpoint of the iliac crest). Raw EMG signals were band pass filtered from 10 to 1000 Hz, amplified (AMT-8, Bortec, Calgary, Canada: CMRR = 115 db at 60 Hz and input impedance = 10 GΩ), and sampled at 4096 Hz with a 16-bit A/D converter (± 2.5 V range). Maximum isometric voluntary contraction (MVC) trials were collected for each muscle against resistance applied by a research assistant (3 trials, 10 s each). For the erector spinae this involved participants extending against resistance with their torso suspended off the end of an examination bench with their lower body fixed [36] and for gluteus medius this involved resisted hip abduction with the subject in the side lying position. A quiet trial was collected with the participant lying prone as a baseline reference for EMG.

#### Spine and pelvic posture

Sagittal thoracic, lumbar, and pelvic angles were calculated from time-varying accelerometer data. The accuracy and reliability of accelerometers to determine spinal angles in the context of sitting has been found to be excellent [37]. The root mean square error of the sensors used in this study was 0.64°. Three tri-axial accelerometers were affixed to the skin with double sided tape in the + y down and + z forward orientation over the following anatomical landmarks: spinous processes of T1, L1 and S1. Accelerometer data were collected continuously in 20-min blocks (2 per 40-min sitting block) and A/D converted using a 16-bit board at a sampling frequency of 4096 Hz. Five normalization trials were collected as follows: quiet standing, full lumbar flexion standing, full lumbar extension standing, full lumbar flexion seated, and full thoracic spine flexion seated.

#### Perceived transient pain

Perceived ratings of transient pain were measured using a digital 100 mm visual analogue scale throughout the study at 10-min intervals. Subjects were asked to rate their pain for the right and left lower back by sliding a bar along a 100 mm continuous line with the following anchors: 0 = no pain whatsoever and 10 = worst pain imaginable using a custom program on their workstation computer (Matlab version R2012b, The MathWorks, Natick, MA, USA). Once ratings were entered the bar reset to zero so past ratings were not viewed again by the participant.

### Data collection protocol

**Fig. 1:**
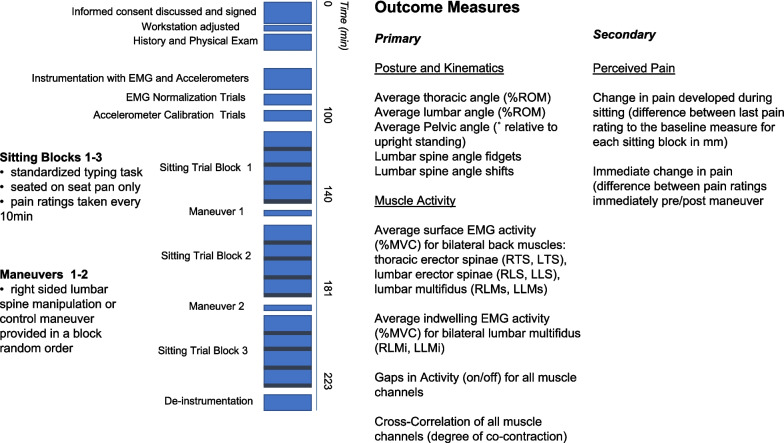
Schematic of the data collection protocol and outcome measures

Figure 1 details the collection protocol and a summary of outcome measures.

#### Preparation

After completing the informed consent process, a brief history, baseline rating of pain, and physical examination of the spine and hips was completed by a licensed chiropractor (8 years of experience) to confirm participants were free of hip/spine pain and that there were no contraindications to SMT. Prior to EMG and accelerometer instrumentation and normalization trials, participants were seated at a computer workstation consisting of height adjustable desk, desktop monitor, keyboard, mouse, and office chair seat pan (regular office chair with the backrest and arm rests removed). Monitor height, keyboard/mouse placement, table height and chair height were adjusted to the participant's anthropometrics and personal preference with reference to ergonomic guidelines [38]. A footrest was used if required.

#### Experimental trial

The 2-h experiment trial involved the participants seated at a computer workstation completing a standardized typing task on the computer with continuous collection of synchronized EMG and accelerometer data. The trial was divided into three 40-min blocks. Perceived pain ratings were taken at the start of each block (time 0) and at 10-min intervals throughout the block. Between blocks 1 and 2, and blocks 2 and 3, in a block randomized order of presentation, participants received either a (1) control maneuver: set up for as for SMT with skin slack tensioned by flexing the top knee and rotating the upper body with a hook contact at the spinous process (practitioner’s fingers contacting the downside/right side of the spinous process) or (2) a high velocity low amplitude (HVLA) SMT set up in the same manner as the control maneuver immediately followed by a HVLA thrust, both centered at the L4/L5 spinous process. Participants were moved from the chair to lying on a portable chiropractic table. The table was placed immediately beside the chair such that participants could transfer by standing up, pivoting, sitting, then lying down on their right side. Participants were blinded to the maneuver order but the researcher delivering the maneuver was not.

### Data reduction

#### Muscle activity

EMG data were processed using Matlab software. EMG signals underwent bias removal, band pass filtering between 30 and 500 Hz, notch filtering with cut-off frequencies of 59–61 Hz, full wave rectification followed by low-pass filtering using a 2nd order Butterworth filter with an effective cut off frequency of 2.5 Hz [39]. Processed signals were then normalized to a percentage of maximum voluntary contraction (% MVC) by subtracting resting EMG levels and dividing by the maximum voluntary contraction (taken as the maximum value of the three MVC trials) for each muscle respectively. Following processing, a gap analysis was conducted to determine the on/off characteristics of each muscle channel. For this, muscle activity at or less than 0.5% MVC for longer than 0.2 s was considered inactive [40, 41]. To assess the degree to which muscle groups were similarly activated cross-correlations of all combinations of muscle pairs were calculated according to the method described by Nelson-Wong et al. [42] using Eq. 1.

1 $${R}_{\mathrm{xy}}\left(\tau \right)= \frac{\frac{1}{T} {\int }_{0}^{T}x\left(t\right)y\left(t+\tau \right)dt}{\sqrt{{R}_{\mathrm{xx}}\left(0\right){R}_{\mathrm{yy}}(0)}}$$

Equation 1 Normalized cross-correlation coefficient *R*_xy_(*τ*) where *x*(*t*) and *y*(*t*) are two signals, *τ* is the phase shift (range ± 1) and *T* is the length of the recording assessed.

Cross-correlations within a window of 500 ms were calculated for each minute of the sitting blocks throughout the study and the absolute maximum *R*_xy_ value was recorded. After confirming no difference between these intervals, the average cross-correlation co-efficient was taken to compare between blocks. Average Normalized EMG and average gap numbers were calculated for each muscle group per block of sitting data.

### Spine and pelvic kinematics

Custom software (Matlab2012, The Mathworks Inc., Natick, Massachusetts, USA) was used to process the accelerometer data by calibrating to gravity, calculating sensor inclination with the arc tan function and then relative angles between the sensors (T1-L1 for the thoracic angle, L1-S2 for the lumbar angle). The pelvic angle was calculated as the inclination of the S2 sensor relative to the vertical. The calibration trials were used to normalize spine angles to the functional range of motion: presenting spine angles as a percentage of maximum flexion range of motion (% ROM) and the pelvic angle relative to upright standing. Movement profiles of the normalized lumbar spine angle was calculated as the frequency and magnitude of fidgets and shifts [42]. Fidgets are classified as small movements of the lumbar angle that quickly move away and back again within a short period of time (seconds). Shifts are longer changes in spine angle that move away from the average baseline and do not return within a short period of time. Outcome measures included average values over the sitting trial. To provide a better idea of the posture distribution found over the entire trial an amplitude probability distribution function (APDF) was calculated for the 10%, 50%, 90% percentiles and range of spine and pelvic angles.

### Perceived transient pain

Custom software was used to record and measure perceived pain throughout the study (Matlab2012, The Mathworks Inc., Natick, Massachusetts, USA). Data was extracted to the nearest mm and the baseline score was subtracted. Since pain and discomfort ratings have been shown to consistently rise throughout prolonged sitting trials for most participants, the last pain score of each block was used for comparison. To assess the immediate impact of the control and manipulation maneuvers the differential between the pain score taken immediately after each maneuver was compared to the last pain score of the preceding sitting block.

### Statistics

The outcome measures included the following factors: normalized spine (thoracic and lumbar) and pelvic angles, spine movement (fidgets, shifts), muscle activity variables (average EMG, gap numbers, and cross-correlation co-efficient per condition) and the last perceived pain score for each condition block. The above variables were compared in a two-way mixed general linear model with sex as a between factor and maneuver type (control and HVLA) as within factors. To compare surface and indwelling lumbar multifidus EMG signals, a two-tailed paired student’s T test was conducted for the right and left pairs of measures from the pre-intervention block only. Statistical significance was accepted at the *p* = 0.05 level and Tukey post hoc testing were completed as required (SAS Statistical Software, version 9.4, SAS Institute Inc, Cary, NC, USA). Effect sizes, calculated as partial eta squared, were interpreted as *η*^2^ = 0.01 small, 0.06 medium, and 0.14 large.

## Results

### Participants

20 participants (10 M/10F) participated in the study. Participant characteristics were as follows: males (average age 24.50 years (SD 6.20), height 1.80 m (SD 0.08) and mass 84.10 kg (SD 20.49) and females (average age 22.00 years (SD 2.71), height 1.65 m (SD 0.06) and mass 63.00 kg (SD 10.79).

### Spine and pelvic posture

**Fig. 2:**
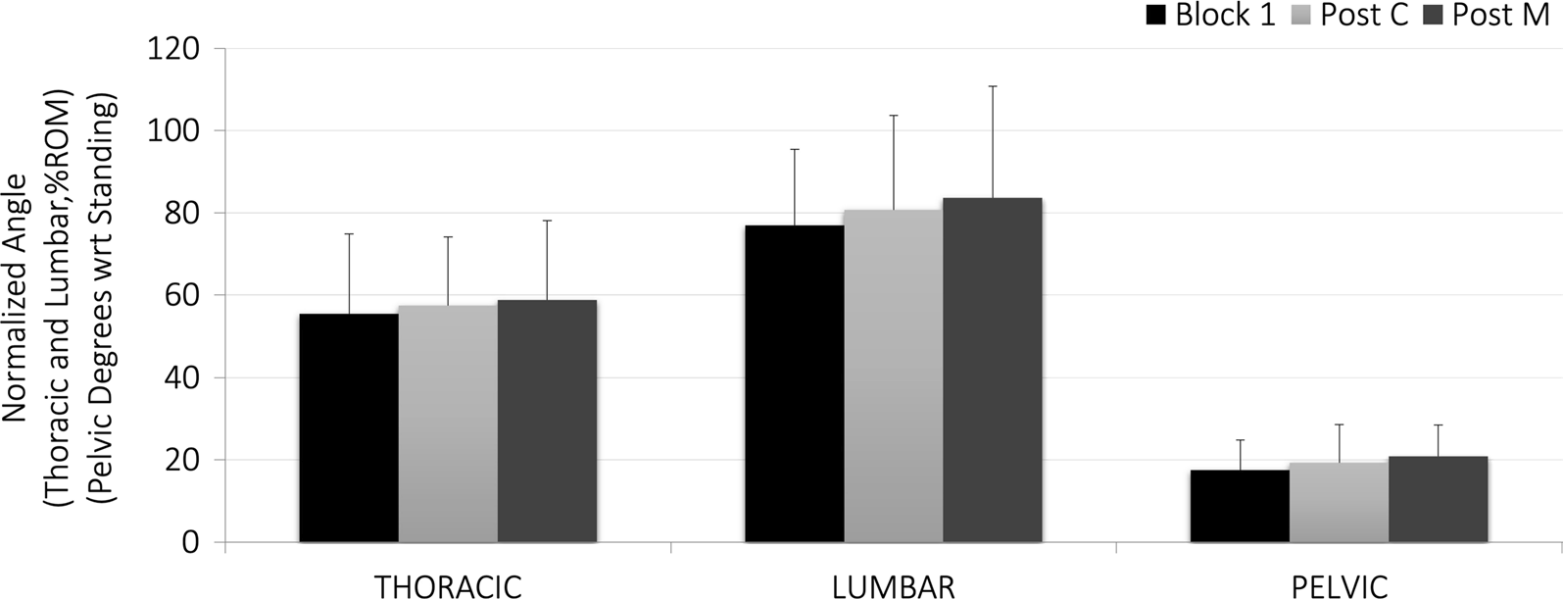
Average normalized thoracic, lumbar, and pelvic flexion angles prior to intervention breaks (block 1) and the sitting blocks following the control (post-C) and HVLA maneuvers (post-M)

**Fig. 3:**
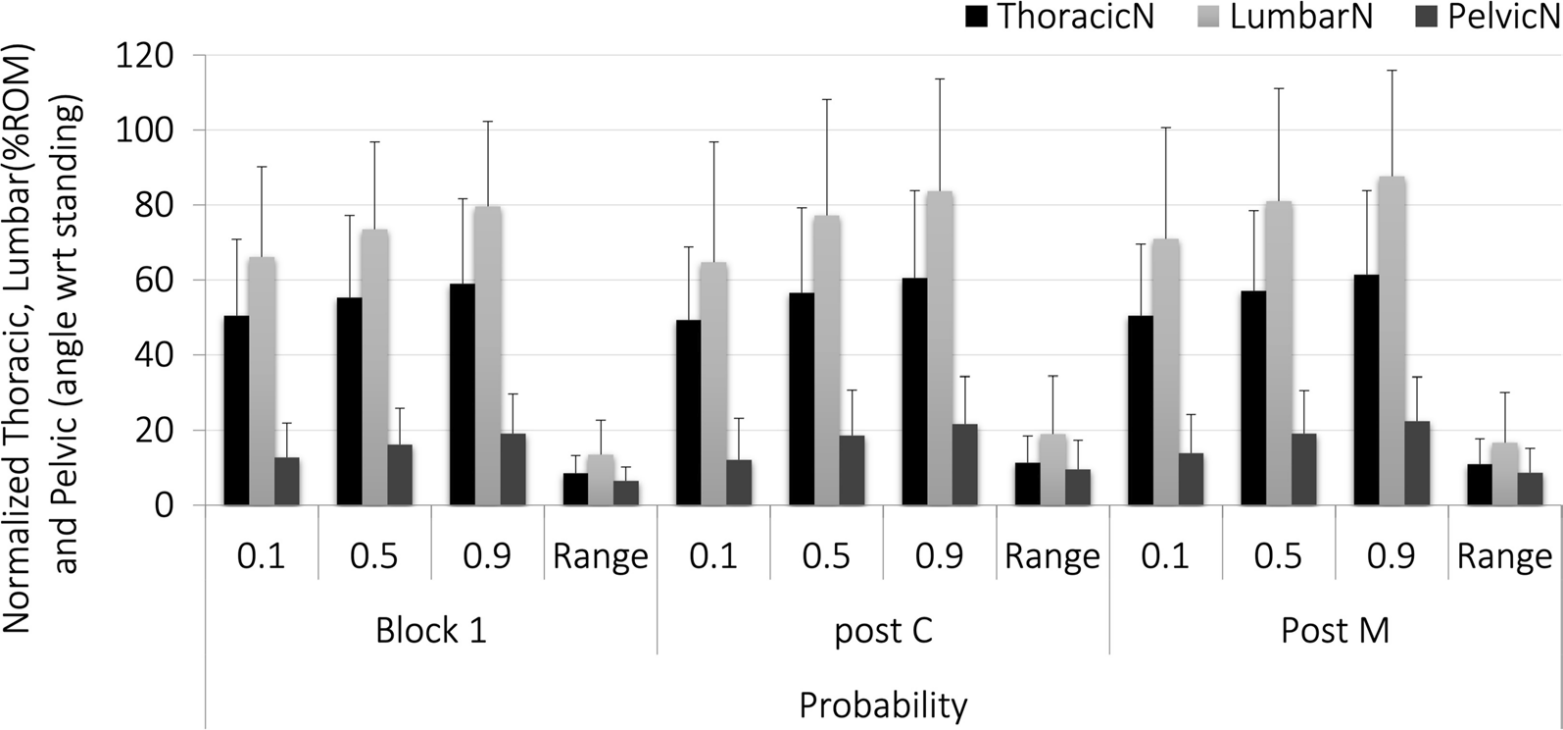
Amplitude Probability Distribution Function (APDF) results for the spine and pelvic angles throughout each sitting block

Participant’s spine (average normalized thoracic and lumbar) and pelvic posture throughout the prolonged sitting blocks were not significantly different in the block proceeding either the control (thoracic 57% ROM SD 17, lumbar 81% ROM SD 23 and pelvic 19° SD 9) or HVLA maneuver (thoracic 59% ROM SD 19, lumbar 84% ROM SD 27 and pelvic 21° SD 8) compared to the baseline sitting block (thoracic 56% ROM SD 19, lumbar 77% ROM SD 18 and pelvic 18° SD 7). There were no significant main effects found for sex or maneuver type for any of these angles and the effect sizes to detect differences between maneuvers was medium for the thoracic angle (*η*^2^ = 0.060) and large for lumbar (*η*^2^ = 0.502) and pelvic (*η*^2^ = 0.631) (Fig. 2). The APDF for these angles throughout each sitting block support the conclusion that subjects sat with these average postures for the majority (90th percentile of probability) of each sitting block (Fig. 3) with a very small range of postures spanning the 10–90th percentiles. From the APDF analysis it was found that the range of postures subjects adopted throughout each block remained small for all angles (thoracic 9–11% ROM, lumbar 13–19% ROM and pelvic 6°–10°, Fig. 3).

### Lumbar angle movements: fidgets and shifts

**Fig. 4:**
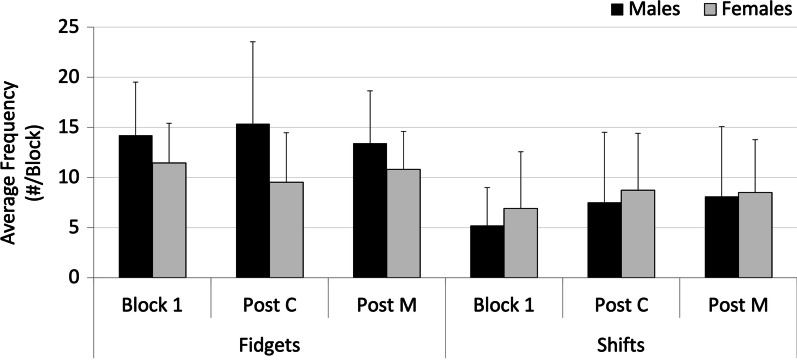
Lumbar spine movement variables: Fidgets (FID) and Shifts averaged over the first block of sitting (baseline) and the sitting blocks following the control (post C) and HVLA (post M) maneuvers for male (black) and female (grey) participants

Within the narrow ranges of spine posture, there were significant differences in the types of movements that were occurring in the lumbar spine. Male subjects moved their lumbar spine more than females. This was reflected in the movement variables. Fidgets, quick movements that return to the original posture, were the dominant type of movement male participants were displayed (significant main effect of sex, *p* = 0.0173). The different types of maneuvers also appeared to influence spine movement. There were significantly greater number of shifts in the post-HVLA sitting block (9 per block SD 0.6) compared to the post-control maneuver block (7 per block SD 1) and baseline block (7 per block SD 2) (*p* = 0.0352, *η*^2^ = 0.851) (Fig. 4). The effect size for detecting differences between conditions was medium for fidgets (*η*^2^ = 0.099) and large for shifts (*η*^2^ = 0.851).

### Muscle Activity

**Fig. 5:**
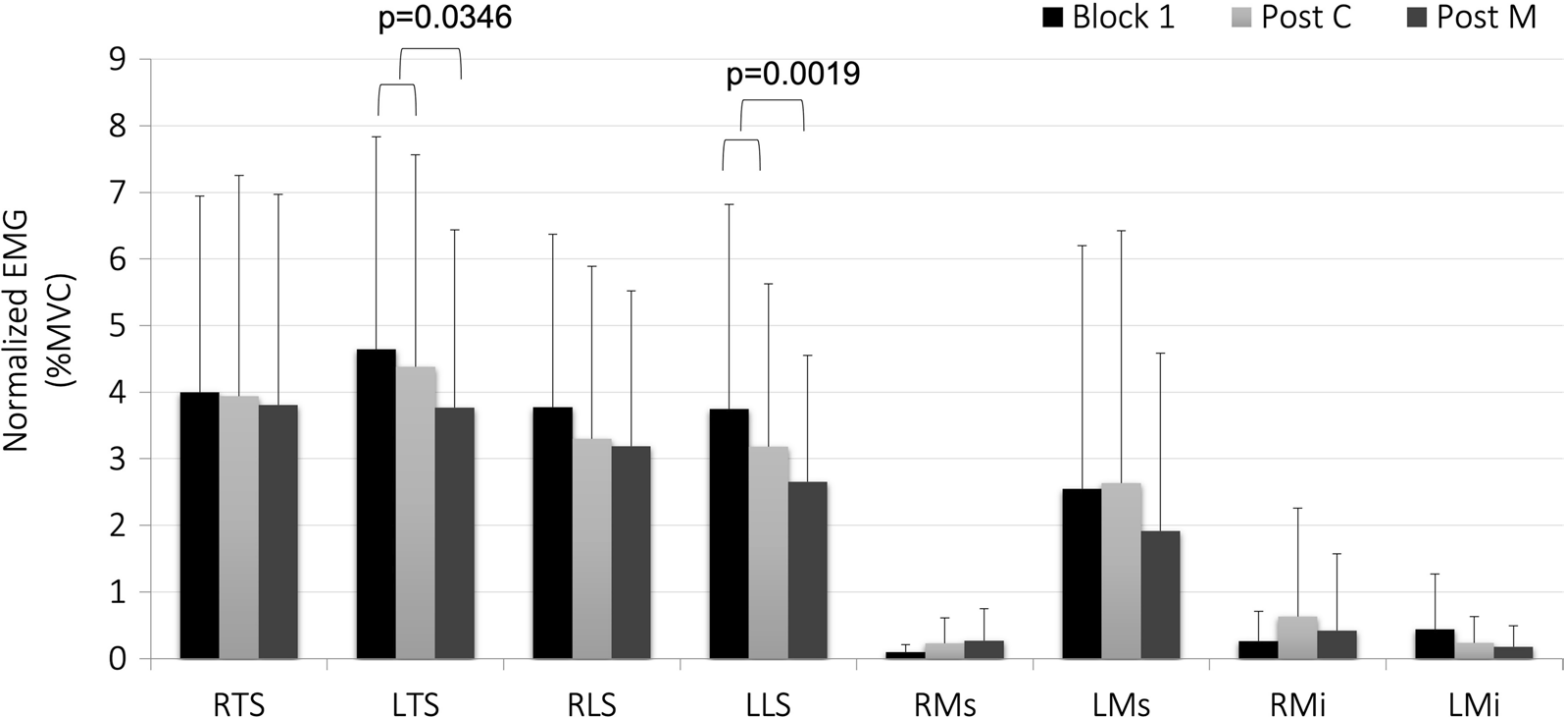
Average EMG (%MVC) for each muscle group in the pre-intervention sitting block (black) and the sitting blocks following the control (light grey) and HVLA (dark grey) maneuvers

Back muscle activity was very low throughout the entire experiment. Average normalized EMG for all muscles did not exceed 4.6%MVC and the range incorporating one standard deviation was maintained under 8%MVC. Effect sizes for detecting differences for the main effects of sex were medium-large for all muscles except for LLS (small: (*η*^2^ = 0.051). Effect sizes for detecting differences for the main effects of condition were large for all muscles except for RTS (small: (*η*^2^ = 0.0425). Generally, higher amounts of activity were found for the thoracic erector spinae, followed by the lumbar erectors, and lumbar multifidii. The average muscle activity of the left thoracic and lumbar erector spinae was significantly lower in the sitting block following the manipulation intervention compared to block 1 and the sitting block following the control maneuver (Fig. 5). There were no significant differences between muscle activity magnitudes between male and females.

**Fig. 6:**
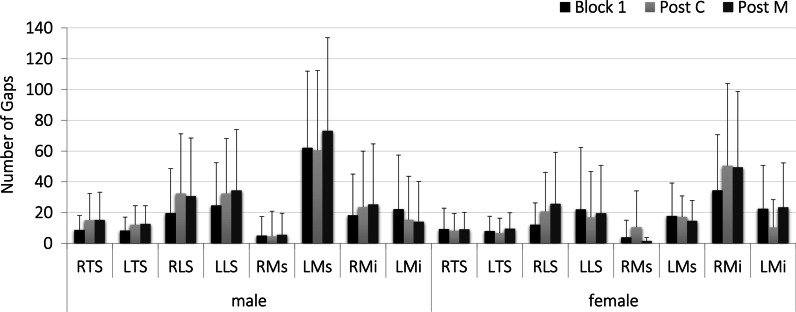
Number of gaps in muscle activity for male and females throughout each prolonged sitting block

Back muscles were not constantly activated throughout each prolonged sitting block. Gaps in activity were documented in all channels throughout the entire collection in both males and female subjects (Fig. 6). A significant 2-way interaction between sex and condition was found for the left lumbar multifidus (LMi) muscle measured with indwelling electrodes (*p* = 0.0416). Gap numbers in male participants dropped from 22 SD 35 in block 1 to 15 SD 28 and 14 SD 26 in the post-C and post-M sitting blocks respectively. Conversely, LMi gap numbers in females dropped from 22 SD 28 in block 1 to 10 SD 18 in the post-C block but increased to 23 SD 49 in the post-M block. A significant main effect of sex was found for the number of gaps occurring in the left lumbar multifidus muscle (surface electrodes). Males displayed a significantly higher number of gaps (65 SD 7) in this muscle throughout all sitting blocks compared to females (17 SD 2, *p* = 0.0078). There were no differences in muscle activity gap numbers between any of the sitting blocks (Fig. 6). The effect sizes for detecting differences in gap numbers between sex and condition were medium-large for all muscles except for LLS (small *η*^2^ = 0.050 for condition), LMs (small *η*^2^ = 0.008 for condition).

**Fig. 7:**
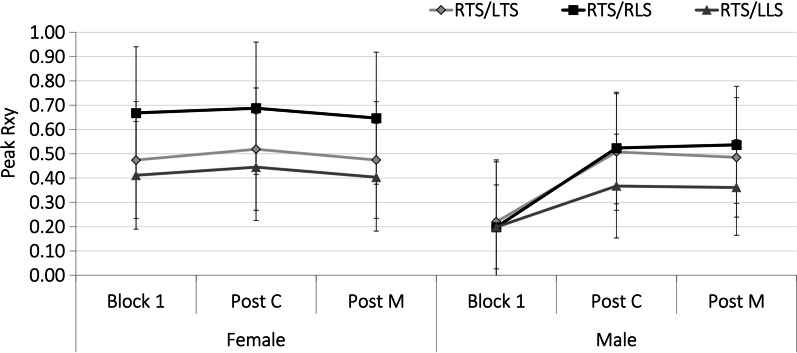
Peak Cross-Correlation co-efficient for muscle combinations throughout each prolonged sitting block for males (right) and females (left). A significant 2-way interaction was found for these pairings

The cross-correlation of EMG signals for pairs of muscles provides an assessment of the degree of co-contraction. Peak cross-correlation coefficients (*R*_xy_) for each muscle combination were compared between sex and sitting blocks. Of the erector spinae and multifidus combinations, significant two-way interactions between sex and sitting block condition were found for the right and left thoracic erector spinae (*p* = 0.0454), right sided thoracic and lumbar erector spinae (*p* = 0.0454) and right thoracic and left lumbar erector spinae (*p* = 0.0400) (Fig. 7). Specifically, for each of these muscle combinations, the degree of correlation stayed relatively the same throughout each block for female subjects but increased between block 1 and the post-C and M blocks for male participants. Generally, the erector spinae and multifidus muscles demonstrate a higher degree of co-contraction than the superficial and deep recordings of multifidus (all combinations having peak *R*_xy_ of less than 0.40 SD 0.25). There were no significant effects of sex or sitting block condition on these 6 combinations of surface and indwelling muscle channels. The effect sizes to detect differences between sex and condition for the cross-correlation variables were medium-large for all combinations except for RTS/LLS (*η*^2^ = 0.045 for condition), LTS/LLS (*η*^2^ = 0.014 for condition), LTS/RLS (*η*^2^ = 0.008 for condition), RLS/RMi (*η*^2^ = 0.048 for condition), LMs/RMs (*η*^2^ = 0.054 for sex), LLS/LMs (*η*^2^ = 0.011 for sex), and LMs/LMi (*η*^2^ = 0.025 for sex).

A comparison of the activity of the superficial and deep portions of the lumbar multifidus (recorded by surface and indwelling electrodes respectively) found a significant difference between the surface and indwelling activity for the left side (surface 2.6% MVC SD 3.7, indwelling 0.4% MVC SD 0.84, *p* = 0.0221). There was no difference between measures taken for the right surface (0.1% MVC SD 0.11) or indwelling signals (0.3% MVC SD 0.45, *p* = 0.1102). The effect size to detect the difference between indwelling and surface channels was large for both the right (*η*^2^ = 0.551) and left (*η*^2^ = 0.955) multifidus muscles.

### Perceived transient back pain

**Fig. 8:**
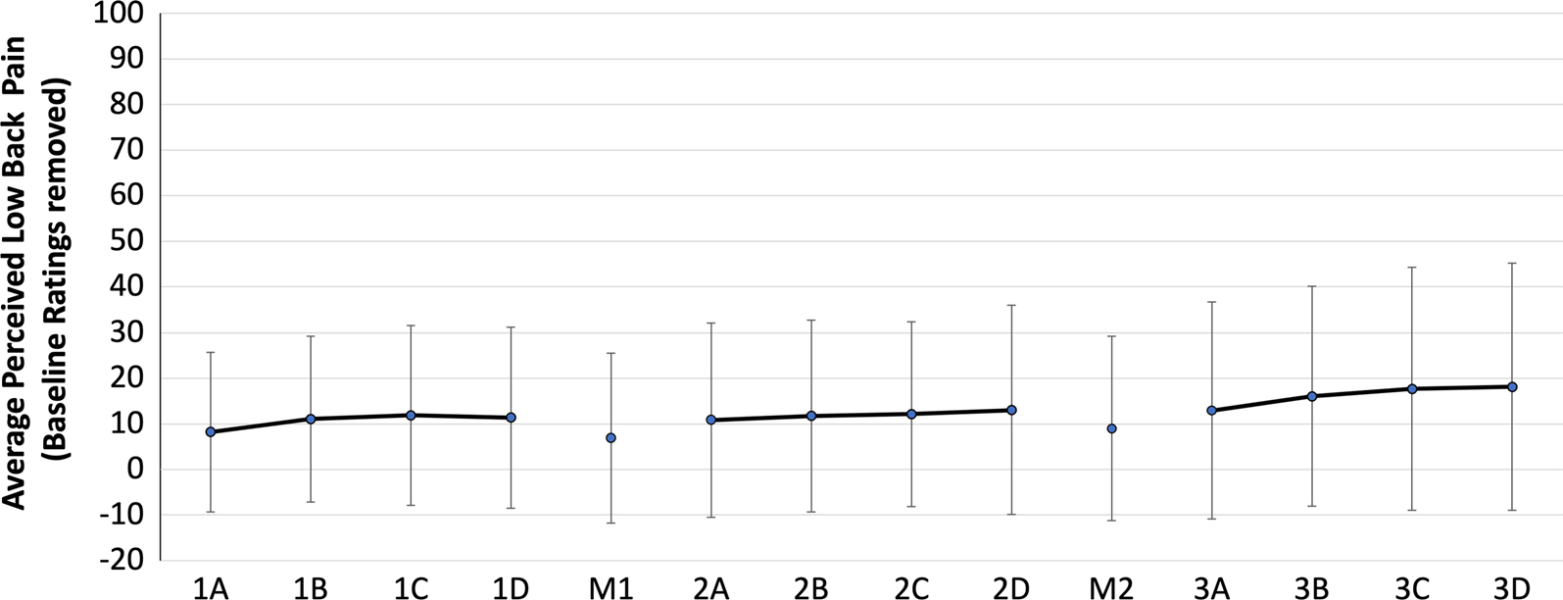
Average perceived low back pain (baseline removed) at 10-min intervals (A-D) throughout each sitting block (1–3). M1 and M2 were the maneuvers delivered in a block randomized order (control = set up for HVLA with pre-load but no thrust, manipulation = setup for HVLA, pre-load, and thrust)

The average baseline pain ratings for the right and left low back were 3 mm ± 8 and 4 mm ± 9 respectively. Thirteen of the twenty participants experienced clinically significant increases in pain from baseline (> 10 mm change on a 100 mm scale [43]) throughout the experiment. There were no significant interactions or main effects for the ultimate transient pain rating (last pain rating of the 40-min sitting block) between sex or maneuver type (control or HVLA) (Fig. 8). Immediate pain reductions following the maneuvers (differential between immediate post-last rating pre) were modest (average improvement of 2.58 ± 8.93 mm and 1.67 ± 7.04 mm for the left and right low back following the control maneuver, and 5.96 ± 10.31 mm and 6.90 ± 11.45 mm for the left and right low back following the manipulation). There were no significant interactions or main effects for sex or maneuver type (control or HVLA) for the differential pain scores for the right or left low back. The effect size for detecting differences for the main effect of sex was large for both the right (*η*^2^ = 0.945) and left (*η*^2^ = 0.770) low back, but small for condition (right (*η*^2^ = 0.034) and left (*η*^2^ = 0.000) low back.

## Discussion

The results of this cross-sectional study suggest that lumbar spinal manipulation does not result in acute changes to spine or pelvic postures adopted during prolonged seated computer work; however, it may influence spine movement parameters and lower muscle activity in a young, healthy population. There were no significant differences in perceived pain immediately following either maneuver or by the end of each 40-min sitting block; however, our observed statistical power to test this difference was low.

### Spine posture and movement

Average thoracic, lumbar, and pelvic angles were not different in any of the three sitting blocks: baseline, post-c (following the control maneuver) or post-m (following HVLA thrust). The magnitude of angles found in this study are within the range found in studies of a similar sitting exposures [44–47], however, it should be noted that with an average lumbar flexion angle of 81% ROM SD 19 and pelvic angle of 19° SD 9 participants did sit at the high range of lumbar flexion (30–80% ROM in unsupported sitting) previously documented [48]. Spine and pelvic postures were no different in the post-c or post-m compared to the baseline sitting block, therefore, the hypothesis that there would be no change in seated spine posture in response to HVLA can be accepted.

Analysis of the time varying signals of low back posture during the prolonged sitting blocks has confirmed that male subjects moved significantly more than females as indicated by the higher fidget frequency, leading to the rejection of the hypothesis that there would be no differences in spine movement between males and females.

From the APDF, lumbar flexion angle ranged between 13 and 19%ROM across all sitting blocks examined in this study indicating that participants adopted a fairly constant posture. Micro movements within this narrow range, however, were identified. Significant differences in shifts were found in the sitting block following the manipulation. Shifts have been associated with increased discomfort in the literature [49]. However, given the small difference between shifts in each of the 3 sitting blocks (between 7 and 9) the likelihood that this difference has practical significance is low. This conclusion is supported by the lack of significant differences found for perceived pain between each of the sitting blocks.

### Muscle activity

Contrary to our hypothesis that no differences in muscle activation levels would be found between conditions, significantly lower levels in low back muscle activity for the left thoracic and lumbar erector spinae muscles were seen in the sitting block following the HVLA maneuver. This unilateral response corresponds to the side of the low back that would be stretched during the procedure (participants were lying down on their right side). While most studies have identified a change in muscle activity following a high-velocity, low-amplitude manipulation, both increases and decreases have been cited [10]. The decreased levels of activation in muscle activity in this study agrees with the findings of previous work [14, 50, 51] but conflicts with the results of some studies using healthy volunteers [17, 52, 53]. Dishman and colleagues have discussed the potential for spinal manipulation to regulate the activation levels of the motoneuronal pool, either increasing or decreasing excitability, which may explain these different effects [54]. Further, passive muscle stretching has been shown to decrease EMG levels in the plantarflexor muscle group [55]. However, methodological factors such as variation in EMG protocol, rate of the manipulation preload, and thrust as well as underlying heterogeneity of the test population could also be factors contributing to the differences found.

Effects of the HVLA maneuver on muscle activity were also evident in muscle activity gap numbers. A differential response was found for the gaps in lumbar multifidus as measured by indwelling electrodes in males and females: activity in this muscle became more constant (fewer number of gaps) in both the post-c and post-m sitting blocks for males but dropped in the post-c block and increased in the post-m block for females. In the surface recording of the left multifidus, male subjects demonstrated a significantly greater number of gaps in activity than females. These results allow the rejection of our hypothesis that there will be no difference in gap numbers between conditions and/or sex. Sex differences in gap number have not been reported previously in published work on sitting [46]. Morl and Bradl [56] have noted that increased lumbar flexion in sitting results in increased gaps in multifidus surface recordings, however, no main effects of sex or condition were found for spine posture in this study. In a recent review of literature, Lehman [10] does conclude that short-term changes in EMG amplitude are associated with spinal manipulation [10]. These differences can most likely be attributed to differences in the maneuver delivery in some way. Theoretically, there should not be any physiological differences between males and females in the response to spinal manipulation. However, given that this is the first study that has examined these EMG parameters in prolonged sitting following manipulation there is no way of assessing the consistency of these results. Future studies may consider investigating these effects with a parallel arm randomized design or a protocol that incorporates a second baseline block.

Both the control maneuver (manipulation set up with pre-load but no thrust) and HVLA (control maneuver plus thrust) appeared to influence the co-contraction of thoracic and lumbar erector spinae muscles in males. Specifically, bilateral thoracic erector spinae, right sided thoracic and lumbar erector spinae and right thoracic and left lumbar erector spinae groups increased the degree of co-contraction in both the post-c and post-m sitting blocks compared to the baseline block. Since the response was similar between the two maneuvers, we must accept our hypothesis, that manipulation would not lead to differences in muscle co-contraction during the sitting exposure. We are also mindful of the low statistical power to detect differences for several of the muscle combinations tested. Studies have shown that the magnitude of preload forces affect the discharge of paraspinal muscle spindles and that increased duration of preload application amplifies this response [57, 58]. Nougarou et al. [53] confirmed the effect of preload parameters on thoracic spine muscles in a population of healthy male and female volunteers aged 20–38. While differences in average EMG activity were only found in response to the manipulation and not the control maneuver in this study, it is possible that the sustained preload alone might have been enough to elicit alterations in relative activation between these muscle groups. Considering that the preload duration was kept the same (5 s) between both the control and manipulation maneuvers, perhaps it is the effect of the preload or induced muscle and joint stretch, and not necessarily the thrust phase, that resulted in the higher co-contraction levels in both the post-c and post-m blocks for male subjects.

### Perceived transient pain

No differences between sex or maneuver type were found between the last pain score of each sitting block (post-m or post-c); however, our observed statistical power to test these differences was low. While the control and HVLA maneuver did result in small immediate decreases in perceived back pain; these were not statistically significant, nor would they be considered clinically significant. Considering all participants received either a preload (control maneuver) or preload plus HVLA thrust to the left side of their low back, the similarity in differential response (and the direction towards improvement) between the right and left sides make sense. There is ample support in the literature for the analgesic effect of spinal manipulation [14, 22, 31, 58–60]. There is also evidence to support that pain inhibition can occur at both the peripheral and central levels of the nervous system, depending on the magnitude and rate of the applied manipulative thrust [22, 58, 61]. Preload parameters have been shown to affect neural responses without a thrust, which may explain the similar result of both the control and manipulation intervention used in this study [58]. A number of groups have documented an analgesic effect from joint mobilizations, where no thrust is present [62–64], and stretching of the low back [65]. The smaller magnitudes of pain reported in this study suggests our data could be limited by a “floor effect”, especially in those individuals that did not develop transient pain during sitting, where there would be no room for further reductions in pain by the maneuvers. Further, lack of significant differences could also be due to the small statistical power and sample size, particularly in that only 13 of the 20 participants experienced clinically relevant changes in pain and might be expected to demonstrate a change.

### Comparison of indwelling and surface recordings of the lumbar multifidus

In this study, significant differences were found between the average surface and indwelling recordings of the lumbar multifidus on the left side, but not the right side of the low back. Levels of activity for all recordings were extremely low, well below 3% MVC even for the relatively “high” left surface channel. Given the high potential for cross talk in surface recordings over the lumbar multifidus [33], a direct comparison of average EMG levels might be misleading. Therefore, to provide a more functional comparison of these signals the peak cross-correlation coefficient (*R*_xy_) for the surface and indwelling signals in block 1 (baseline) was compared for each side. The left surface/indwelling combination had a peak *R*_xy_ of 0.39 SD 0.04 and the right combination had a peak *R*_xy_ of 0.23 SD 0.04. Both coefficients are low enough to conclude that these muscle portions are functionally different, despite the lack of significant difference in average activity between the surface and indwelling recordings on the left side. This conclusion agrees with the work of Stokes and colleagues [33], who also found low correlation between superficial and indwelling multifidus recordings that supports the conclusion of differential function between the superficial and deep portions of the muscle proposed by Moseley et al. [66].

### Limitations

This study represents the first investigation on the effect of spinal manipulation on biomechanical factors and perceived pain in prolonged office sitting. The results of this work suggest that spinal manipulation influences muscle activity and spine movement during prolonged sitting, however, there are several limitations that must be considered. Although most research in the area of spinal manipulation confirms the extremely short-lived effects [9, 27, 50, 52, 61], the design of this study did not provide a washout period for potential carry-over effects between the control or manipulation maneuvers.

Subjects were not blinded to either the control or manipulation maneuvers; however, they were not told which order the interventions would be presented. For the participants that have received a spinal manipulation in the past, they would be able to tell when a manipulation versus control maneuver was delivered. However, the primary purpose of this study was the effect on biomechanical parameters of posture and muscle activity, variables that are less likely to be influenced by placebo. Order was not originally tested in this analysis as it was assumed that block randomization would successfully avert this effect. Post hoc order testing could not be completed since order was not linked to subject numbers in our database. Knowledge of maneuver type might have been expected, however, to affect the perceived pain ratings immediately after the interventions were performed, which were not different in this case. Therefore, we are confident that the lack of blinding had minimal impact on our results.

The participants involved in this study were young and healthy, the study population was small, and the proportion of participants that developed clinically relevant increases in perceived pain was smaller (13/20). Spinal manipulation is a therapy that is used to treat biomechanical lesions such as motion segment hypomobility, pain and muscle spasm [67]. Investigating the effect of this intervention in an asymptomatic population has been raised as a potential limitation, perhaps minimizing effects that would be seen otherwise in a clinical population [68]. However, there have been studies that have found physiological effects of manipulation in healthy populations and animal models without the presence of these lesions, which does lend support for the use of this population as a starting point before moving to clinical groups [9, 50, 52, 58, 61, 68]. The intensive instrumentation and data processing involved in this study limited the sample size due to resources. While sufficient effect sizes were obtained for most primary biomechanical outcome measures in this study, the ability to detect differences between conditions for perceived pain was poor; therefore, conclusions related to pain in this study should be interpreted cautiously. The results of this study should be replicated with a larger sample, ideally in a field setting, with a more generalizable population.

## Conclusion

The results of this study suggest that spinal manipulation may play a role in increasing low back movement parameters and lowering muscle activity during sitting in a young, healthy population. Since there were no differences in the spine or pelvic postures adopted throughout this study, it can be concluded that spinal manipulation does not appear to have an immediate impact on spine posture in sitting in young, asymptomatic individuals. Future work should replicate these results with a larger, more generalized sample in a field setting. It may be worthwhile to explore the implication of reduced muscle activation and increased spine movements during prolonged sitting to for office workers that receive manipulations or mobilizations during their workday.

## Data Availability

The dataset used in this study are available from the corresponding author on reasonable request.

## Abbreviations

% MVC: Percent maximum voluntary contraction
% ROM: Percent range of motion
A/D: Analog to digital
ANOVA: Analysis of variance
APDF: Amplitude probability distribution function
C: Control maneuver
EMG: Electromyography
FID: Fidgets
HVLA: High velocity low amplitude
L4: 4th lumbar
L5: 5th lumbar
LBP: Low back pain
LLS: Left lumbar erector spinae
LMi: Left lumbar multifidus indwelling
LMs: Left lumbar multifidus surface
LTS: Left thoracic erector spinae
M: Manipulation maneuver
RLS: Right lumbar erector spinae
RMi: Right lumbar multifidus indwelling
RMs: Right lumbar multifidus surface
RTS: Right thoracic erector spinae
Rxy: Cross-correlation co-efficient
S2: 2nd Sacral
SD: Standard deviation
SMT: Spinal manipulative therapy

## References

[1] Chou R, Huffman LH, American Pain Society, American College of Physicians (2007). Nonpharmacologic therapies for acute and chronic low back pain: a review of the evidence for an American Pain Society/American College of Physicians clinical practice guideline. Ann Intern Med.

[2] Dagenais S, Tricco AC, Haldeman S (2010). Synthesis of recommendations for the assessment and management of low back pain from recent clinical practice guidelines. Spine J.

[3] Bussieres AE, Stewart G, Al-Zoubi F, Decina P, Descarreaux M, Haskett D (2018). Spinal manipulative therapy and other conservative treatments for low back pain: a guideline from the canadian chiropractic guideline initiative. J Manip Physiol Ther.

[4] Eklund A, Hagberg J, Jensen I, Leboeuf-Yde C, Kongsted A, Lövgren P (2020). The Nordic maintenance care program: maintenance care reduces the number of days with pain in acute episodes and increases the length of pain free periods for dysfunctional patients with recurrent and persistent low back pain—a secondary analysis of a pragmatic randomized controlled trial. Chiropr Man Therap.

[5] Beliveau PJH, Wong JJ, Sutton DA, Simon NB, Bussières AE, Mior SA (2017). The chiropractic profession: a scoping review of utilization rates, reasons for seeking care, patient profiles, and care provided. Chiropr Man Therap.

[6] Bishop MD, Beneciuk JM, George SZ (2011). Immediate reduction in temporal sensory summation after thoracic spinal manipulation. Spine J.

[7] Bronfort G, Haas M, Evans RL, Bouter LM (2004). Efficacy of spinal manipulation and mobilization for low back pain and neck pain: a systematic review and best evidence synthesis. Spine J.

[8] Colloca CJ, Keller TS (2007). Spinal manipulation reduces pain and hyperalgesia after lumbar intervertebral foramen inflammation in the rat. J Manip Physiol Ther.

[9] Herzog J (1999). Use of cervical spine manipulation under anesthesia for management of cervical disk herniation, cervical radiculopathy, and associated cervicogenic headache syndrome. J Manip Physiol Ther.

[10] Lehman GJ, McGill SM (2001). Spinal manipulation causes variable spine kinematic and trunk muscle electromyographic responses. Clin Biomech.

[11] Mansilla-Ferragut P, Fernandez-de-Las Penas C, Alburquerque-Sendin F, Cleland JA, Bosca-Gandia JJ (2009). Immediate effects of atlanto-occipital joint manipulation on active mouth opening and pressure pain sensitivity in women with mechanical neck pain. J Manip Physiol Ther.

[12] Taylor HH, Murphy B (2010). Altered central integration of dual somatosensory input after cervical spine manipulation. J Manipul Physiol Ther.

[13] Passmore SR, Burke JR, Good C, Lyons JL, Dunn AS (2010). Spinal manipulation impacts cervical spine movement and Fitts’ task performance: a single-blind randomized before-after trial. J Manip Physiol Ther.

[14] Bicalho E, Palma Setti JA, Macagnan J, Rivas Cano JL, Manffra EF (2010). Immediate effects of a high-velocity spine manipulation in paraspinal muscles activity of nonspecific chronic low-back pain subjects. Man Ther.

[15] Gill NW, Teyhen DS, Lee IE (2007). Improved contraction of the transversus abdominis immediately following spinal manipulation: a case study using real-time ultrasound imaging. Man Ther.

[16] Keller TS, Colloca CJ (2000). Mechanical force spinal manipulation increases trunk muscle strength assessed by electromyography: a comparative clinical trial. J Manip Physiol Ther.

[17] Lalanne K, Lafond D, Descarreaux M (2009). Modulation of the flexion-relaxation response by spinal manipulative therapy: a control group study. J Manip Physiol Ther.

[18] Suter E, McMorland G, Herzog W, Bray R (1999). Decrease in quadriceps inhibition after sacroiliac joint manipulation in patients with anterior knee pain. J Manip Physiol Ther.

[19] Suter E, McMorland G, Herzog W (2005). Short-term effects of spinal manipulation on H-reflex amplitude in healthy and symptomatic subjects. J Manipul Physiol Ther.

[20] Triano JJ (2001). Biomechanics of spinal manipulative therapy. Spine J.

[21] Haavik H, Murphy B (2011). Subclinical neck pain and the effects of cervical manipulation on elbow joint position sense. J Manip Physiol Ther.

[22] Haavik-Taylor H, Murphy B (2007). Cervical spine manipulation alters sensorimotor integration: a somatosensory evoked potential study. Clin Neurophysiol.

[23] Morningstar MW, Strauchman MN, Weeks DA (2003). Spinal manipulation and anterior headweighting for the correction of forward head posture and cervical hypolordosis: a pilot study. J Chiropr Med.

[24] Palmgren PJ, Lindeberg A, Nath S, Heikkila H (2009). Head repositioning accuracy and posturography related to cervical facet nerve blockade and spinal manipulative therapy in healthy volunteers: a time series study. J Manip Physiol Ther.

[25] Rogers RG (1997). The effects of spinal manipulation on cervical kinesthesia in patients with chronic neck pain: a pilot study. J Manip Physiol Ther.

[26] Sung PS, Kang YM, Pickar JG (2005). Effect of spinal manipulation duration on low threshold mechanoreceptors in lumbar paraspinal muscles: a preliminary report. Spine (Phila Pa1976)..

[27] Herzog W (2010). The biomechanics of spinal manipulation. JBodywork Movement Ther.

[28] McGill SM, Hughson RL, Parks K (2000). Lumbar erector spinae oxygenation during prolonged contractions: implications for prolonged work. Ergonomics.

[29] Solomonow M, Zhou BH, Baratta RV, Burger E (2003). Biomechanics and electromyography of a cumulative lumbar disorder: response to static flexion. ClinBiomech.

[30] Korakakis V, O’Sullivan K, Kotsifaki A, Sotiralis Y, Giakas G. Lumbo-pelvic proprioception in sitting is impaired in subgroups of low back pain–But the clinical utility of the differences is unclear. A systematic review and meta-analysis. PLoS ONE [Internet]. 2021;16(4 April). https://www.scopus.com/inward/record.uri?eid=2-s2.0-85104893997&doi=10.1371%2fjournal.pone.0250673&partnerID=40&md5=2fae74c5480806f321db81b8c4330484 10.1371/journal.pone.0250673 PMC8075231 33901255

[31] Vining R, Long CR, Minkalis A, Gudavalli MR, Xia T, Walter J (2020). Effects of chiropractic care on strength, balance, and endurance in active-duty U.S. Military Personnel with Low Back Pain: A randomized controlled trial. J Altern Complement Med.

[32] Fagundes Loss J, de Souza da Silva L, Ferreira Miranda I, Groisman S, Santiago Wagner Neto E, Souza C (2020). Immediate effects of a lumbar spine manipulation on pain sensitivity and postural control in individuals with nonspecific low back pain: a randomized controlled trial. Chiropr Man Therap..

[33] Stokes IAF, Henry SM, Single RM (2003). Surface EMG electrodes do not accurately record from lumbar multifidus muscles. ClinBiomech.

[34] Stokes M, Rankin G, Newham DJ (2005). Ultrasound imaging of lumbar multifidus muscle: normal reference ranges for measurements and practical guidance on the technique. ManTher.

[35] Blouin JS, Siegmund GP, Carpenter MG, Inglis JT (2007). Neural control of superficial and deep neck muscles in humans. JNeurophysiol.

[36] Dankaerts W, O’Sullivan PB, Burnett AF, Straker LM, Danneels LA (2004). Reliability of EMG measurements for trunk muscles during maximal and sub-maximal voluntary isometric contractions in healthy controls and CLBP patients. J Electromyogr Kinesiol.

[37] Wong WY, Wong MS (2008). Detecting spinal posture change in sitting positions with tri-axial accelerometers. Gait Posture.

[38] Canadian Standards Association Group. Office Ergonomics—an application standard for workplace ergonomics. No. Z412–17. 2017.

[39] Brereton LC, McGill SM (1998). Frequency response of spine extensors during rapid isometric contractions: effects of muscle length and tension. J Electromyogr Kinesiol.

[40] Gregory DE, Callaghan JP (2008). Prolonged standing as a precursor for the development of low back discomfort: an investigation of possible mechanisms. Gait Posture.

[41] Veiersted KB, Westgaard RH, Andersen P (1993). Electromyographic evaluation of muscular work pattern as a predictor of trapezius myalgia. Scand J Work Environ Health.

[42] Gallagher KM, Nelson-Wong E, Callaghan JP (2011). Do individuals who develop transient low back pain exhibit different postural changes than non-pain developers during prolonged standing?. Gait Posture.

[43] Olsen MF, Bjerre E, Hansen MD, Hilden J, Landler NE, Tendal B (2017). Pain relief that matters to patients: systematic review of empirical studies assessing the minimum clinically important difference in acute pain. BMC Med.

[44] Beach TA, Mooney SK, Callaghan JP (2003). The effects of a continuous passive motion device on myoelectric activity of the erector spinae during prolonged sitting at a computer workstation. Work.

[45] Dunk NM, Callaghan JP (2005). Gender-based differences in postural responses to seated exposures. Clin Biomech (Bristol, Avon)..

[46] Gregory DE, Dunk NM, Callaghan JP (2006). Stability ball versus office chair: comparison of muscle activation and lumbar spine posture during prolonged sitting. Hum Factors.

[47] Greene RD, Frey M, Attarsharghi S, Snow JC, Barrett M, Carvalho D (2019). Transient perceived back pain induced by prolonged sitting in a backless office chair: are biomechanical factors involved?. Ergonomics.

[48] Callaghan JP, McGill SM (2001). Low back joint loading and kinematics during standing and unsupported sitting. Ergonomics.

[49] Dunk NM, Callaghan JP (2010). Lumbar spine movement patterns during prolonged sitting differentiate low back pain developers from matched asymptomatic controls. Work.

[50] DeVocht JW, Pickar JG, Wilder DG (2005). Spinal manipulation alters electromyographic activity of paraspinal muscles: a descriptive study. J Manip Physiol Ther.

[51] Lehman GJ, McGill SM (1999). The influence of a chiropractic manipulation on lumbar kinematics and electromyography during simple and complex tasks: a case study. J Manip Physiol Ther.

[52] Nougarou F, Dugas C, Deslauriers C, Pagé I, Descarreaux M (2013). Physiological responses to spinal manipulation therapy: investigation of the relationship between electromyographic responses and peak force. J Manip Physiol Ther.

[53] Nougarou F, Dugas C, Loranger M, Pagé I, Descarreaux M (2014). The role of preload forces in spinal manipulation: experimental investigation of kinematic and electromyographic responses in healthy adults. J Manip Physiol Ther.

[54] Dishman JD, Greco DS, Burke JR (2008). Motor-evoked potentials recorded from lumbar erector spinae muscles: a study of corticospinal excitability changes associated with spinal manipulation. J Manip Physiol Ther.

[55] Ryan ED, Herda TJ, Costa PB, Herda AA, Cramer JT (2014). Acute effects of passive stretching of the plantarflexor muscles on neuromuscular function: the influence of age. Age (Dordr).

[56] Morl F, Bradl I (2013). Lumbar posture and muscular activity while sitting during office work. J Electromyogr Kinesiol.

[57] Dishman JD, Dougherty PE, Burke JR (2005). Evaluation of the effect of postural perturbation on motoneuronal activity following various methods of lumbar spinal manipulation. Spine J.

[58] Reed WR, Sozio R, Pickar JG, Onifer SM (2014). Effect of spinal manipulation thrust duration on trunk mechanical activation thresholds of nociceptive-specific lateral thalamic neurons. J Manip Physiol Ther.

[59] Maduro de Camargo V, Alburquerque-Sendín F, Bérzin F, Cobos Stefanelli V, Rodrigues de Souza DP, Fernández-de-las-Peñas C (2011). Immediate effects on electromyographic activity and pressure pain thresholds after a cervical manipulation in mechanical neck pain: a randomized controlled trial. J Manip Physiol Ther..

[60] de Zoete A, Rubinstein SM, de Boer MR, Ostelo R, Underwood M, Hayden JA (2021). The effect of spinal manipulative therapy on pain relief and function in patients with chronic low back pain: an individual participant data meta-analysis. Physiotherapy.

[61] Pickar JG, Bolton PS (2012). Spinal manipulative therapy and somatosensory activation. J Electromyogr Kinesiol.

[62] Gross A, Miller J, D’Sylva J, Burnie SJ, Goldsmith CH, Graham N (2010). Manipulation or mobilisation for neck pain: a cochrane review. Man Ther.

[63] Krouwel O, Hebron C, Willett E (2010). An investigation into the potential hypoalgesic effects of different amplitudes of PA mobilisations on the lumbar spine as measured by pressure pain thresholds (PPT). Man Ther.

[64] Willett E, Hebron C, Krouwel O (2010). The initial effects of different rates of lumbar mobilisations on pressure pain thresholds in asymptomatic subjects. Man Ther.

[65] Sayson JV, Hargens AR (2008). Pathophysiology of low back pain during exposure to microgravity. Aviat Space Environ Med.

[66] Moseley GL, Hodges PW, Gandevia SC (2002). Deep and superficial fibers of the lumbar multifidus muscle are differentially active during voluntary arm movements. Spine (Phila Pa1976)..

[67] Henderson CNR (2012). The basis for spinal manipulation: chiropractic perspective of indications and theory. J Electromyogr Kinesiol.

[68] Cao DY, Reed WR, Long CR, Kawchuk GN, Pickar JG (2013). Effects of thrust amplitude and duration of high-velocity, low-amplitude spinal manipulation on lumbar muscle spindle responses to vertebral position and movement. J Manip Physiol Ther.

